# Responses of soil enzymatic activities and microbial biomass phosphorus to improve nutrient accumulation abilities in leguminous species

**DOI:** 10.1038/s41598-024-61446-z

**Published:** 2024-05-15

**Authors:** Farheen Solangi, Xingye Zhu, Kashif Ali Solangi, Rashid Iqbal, Mohamed S. Elshikh, Khaloud Mohammed Alarjani, Heba H. Elsalahy

**Affiliations:** 1 Research Centre of Fluid Machinery Engineering and Technology, Jiangsu University, Zhenjiang, 212013 China; 2 Key Laboratory of Modern Agricultural Equipment and Technology, Ministry of Education, Institute of Agricultural Engineering, Jiangsu University, Zhenjiang, 212013 Jiangsu China; 3 Department of Agronomy, Faculty of Agriculture and Environment, The Islamia University of Bahawalpur, Bahawalpur, 63100 Pakistan; 4 Department of Botany and Microbiology, College of Science, King Saud University, P.O. 2455, 11451 Riyadh, Saudi Arabia; 5 Leibniz Centre for Agricultural Landscape Research (ZALF), 15374 Müncheberg, Germany

**Keywords:** Fertilization techniques, Legumes species, Soil enzymes, Nutrient uptake, Microbial biomass, Phosphorus, Potassium, Biological techniques, Plant sciences

## Abstract

Fertilizers application are widely used to get a higher yield in agricultural fields. Nutrient management can be improved by cultivating leguminous species in order to obtain a better understanding of the mechanisms that increase the amount of available phosphorus (P) and potassium (K) through fertilizer treatments. A pot experiment was conducted to identify the leguminous species (i.e., chickpea and pea) under various fertilizer treatments. Experimental design is as follows: T0 (control: no fertilizer was applied), T1: P applied at the level of (90 kg ha^−1^), T2: (K applied at the level of 90 kg ha^−1^), and T3: (PK applied both at 90 kg ha^−1^). All fertilizer treatments significantly (*p* < 0.05) improved the nutrient accumulation abilities and enzymes activities. The T3 treatment showed highest N uptake in chickpea was 37.0%, compared to T0. While T3 developed greater N uptake in pea by 151.4% than the control. However, T3 treatment also increased microbial biomass phosphorus in both species i.e., 95.7% and 81.5% in chickpeas and peas, respectively, compared to T0 treatment. In chickpeas, T1 treatment stimulated NAGase activities by 52.4%, and T2 developed URase activities by 50.1% higher than control. In contrast, T3 treatment enhanced both BGase and Phase enzyme activities, i.e., 55.8% and 33.9%, respectively, compared to the T0 treatment. Only the T3 treatment improved the activities of enzymes in the pea species (i.e., BGase was 149.7%, URase was 111.9%, Phase was 81.1%, and NAGase was 70.0%) compared to the control. Therefore, adding combined P and K fertilizer applications to the soil can increase the activity of enzymes in both legume species, and changes in microbial biomass P and soil nutrient availability make it easier for plants to uptake the nutrients.

## Introduction

Nutrient management is an important strategy for achieving high plant yield and maintaining soil fertility status^1^. Nitrogen (N) Phosphorus (P) and potassium (K) are the primary macronutrients that regulate the plant growth and development^2^. These essential nutrients play a significant role in physiological processes, such as N, which is directly related to the photosynthesis process, which is important for the healthy vegetative growth of a plant. Phosphorus and K are involved in protein synthesis, enzyme activation, glycolysis, and sugar transport redox reactions^3,4^. Macronutrient deficiencies in agriculture are widespread, affecting plant production around the world^5^. Phosphorus and K fertilizers will continue to play a vital role in agricultural systems. It turns out that any disturbance, including fertilization, crop rotation, tillage, or pollution, primarily affects this organic layer of soil and affects the microbial communities^6,7^. Pakistan is situated in an arid to semi-arid area of the world, and deficiency of P is mainly noted in soils that have lower moisture contents and dry lands^8^. About 80–90% of Pakistani soils are P-deficient due to their high carbonate content (CaCO_3_ > 3.0%) and alkaline pH > 7.0. There is a need to improve its range to supply external P content^9^.

The application of fertilizer could increase several processes; the first would modify the properties of the soil^10^. However, diminishing natural resources in P and K are a serious concern due to reduced availability and increased costs^11,12^. Fertilizer application, tillage practices, and other conventional agricultural methods alter soil properties, requiring the improvement and examination of soil quality^13,14^. Therefore, the application of extensive fertilizer not only increases the P fixation capacity but also causes environmental pollution. In advance agriculture, inorganic and organic fertilizer, or a combination of both, were used to get higher production. On the other hand, biological processes have significance for the functioning of agricultural systems^15^. Soil microbes regulate P cycling through organic phosphate mobilization and the breakdown of organic matter. Soil microbial biomass, also known as microbiological biomass P (MBP), can accumulate released P. Soil microbes and plants may benefit from MBP's multiple functions as a P sink and the accumulation of potentially bioavailable P. Microbes in the soil compete with plants for plant-available P^16^. Green manures are a fast-growing crop that is one of the most effective methods for improving soil quality in a variety of ways. They have the ability to enhance soil quality, organic matter, and improve nutrient availability, such as P and K. In this regard, the present study cultivates leguminous species to find a better understanding of the mechanisms that improve plant nutrients and fertilizer treatments. Leguminous green manure crops have the potential to improve the nitrogen cycle of the soil and highly absorb nutrients from the soil, and high nutrient uptake capacity is mainly related to plant root morphological traits to achieve high yields under low nutrient conditions because a plant may use different strategies to take up P and K from low-nutrient soils^17^. Leguminous plants can produce pod-shaped fruits that contain fleshy seeds that are known as beans.

In Pakistan, peas and chickpeas are important food legume crops. These are significant nutritional legume crops that provide a low-cost, necessary supply of protein. Chickpeas are a valuable source of vitamins, carbohydrates, and minerals. Both chickpeas and peas are multi-functional legume crops that play an important role in small-scale economics for farmers^18^. Leguminous plants can easily acquire greater amounts of P and K from the soil for their growth and the associated root morphological mechanisms^19,20^. Plant root characteristics can improve soil aggregates, decrease soil bulk density, and maintain soil pH; these factors can possibly increase soil quality^21^. The root systems of leguminous species are mainly occupied by a large number of microorganisms that also have the ability to release enzymes, which contribute significantly to the various processes that are associated with plant growth^22,23^. Soil microorganisms perform the basic role in soil organic P transformation, and microbial biomass is also essential for nutrient cycling in soil status^24^. It is assumed that the microbial biomass P is directly released from cells when microbes die, is certainly decomposed in soil, and can be easily taken by plants^12,25^. Soil enzymatic activities are an indicator of microbial activity and function. They can modify soil chemical processes, and soil organic matter (SOM) dynamics contribute to changes in abiotic and biotic factors in soil^26,27^. In order to examine the soil microbial biomass,^28^ reported that fertilizer treatments also had an impact on a number of enzyme activities^29^. Each enzyme plays a specific role in increasing nutrient availability, while some enzymes related to the carbon cycle (C), for example, β-xylosidase and β-glucosidase are famous for their quick responses^30^. However, urease (URase) and N-acetylglucosaminidase (NAGase) enzymes that regulate the N cycle hydrolyze urea into ammonia and carbon dioxide, and NAGase breakdown amino acids into sugar, which are the main sources of N mineralization^31^. Some enzymes are famous for their quick responses, such as β-xylosidase and β-glucosidase. These enzymes break down complex molecules into simple ones for plant uptake^32^. It was previously reported that phosphatase activity catalyzes the cleavage of P minerals from organic phosphates in acidic and alkaline soils and enhances organic matter with the help of mycorrhizal species^33,34^.

An estimation of the amount of soil available as nutrients for crops would help in the design of fertilizer application regimes to optimize the P and K fertilization rates. By measuring the amount of P and K present in the soil, it is possible to estimate the fertilizer application rate^35^^,^^36,37^. Many researchers have been emphasizing the N fertilizer treatment to evaluate the leguminous species, further demonstrating the activities of enzymes affected by N fertilization^38,39^. An earlier study used mineral fertilizer applications with manures and N fertilizer applications to investigate the responses of enzymatic activities to legume crops^40^. Keeping this view in mind, the present research focuses on appropriate P and K fertilizer strategies and responses to improve nutrient uptake and nutrient use efficiency in legume species and their impact on soil nutrient availability. In addition, there is limited research on how soil enzymes and microbial biomass phosphorus vary in response to the cultivation of leguminous species under different P and K fertilizer treatments. It is hypothesized that specific fertilizer treatments could potentially improve the mechanisms involved in plant nutrient uptake and plant nutrient use efficiency subsequently increasing the dry biomass yield of leguminous crops. This study aims to investigate the responses of soil enzymes (such as β-glucosidase phosphatase, Urease, and N-acetylglucosaminidase) and microbial biomass phosphorus to variations in PK fertilization treatments. Further, explore how these responses showed a correlation with the nutrient uptake abilities of legume crops.

## Materials and methodology

### Experimental design

The pot experiment was carried out in Sindh province, located between coordinates 25°42 34″ N and 68°54′ 08″ E in southern Pakistan, to determine the responses of two different legume species under P and K fertilizer techniques. We collected the experimental soil from the field of the Pulses Research Sub-station, Tandojam Sindh, using an auger with an internal diameter of 5 cm and a depth of 0–20 cm. The basic soil properties are the following: soil organic matter (SOM) 0.65%; total N (TN) 1.5 g kg^−1^; available P 10.8 mg kg^−1^ and K 112.3 mg kg^−1^; soil pH 7.8 (1:2.5), EC was 0.35 cmol kg^−1^ and CaCO_3_ was 8.3% with clay loam texture class. While the seeds of legume species were collected from the Agriculture Research Institute in Tandojam. An experiment design was planned as a completely randomized design with factorial arrangements; each treatment had four replications, and a total of 16 pots were used for each legume species. Every pot was manually packed with 7 kg of dry soil with upper 28 cm diameters and lower 18 cm and a depth of 26 cm with a surface area of 0.049 m^2^. However, each pot was properly homogenized with P (P_2_O_5_), and K (K_2_O) fertilizers.

**Figure 1:**
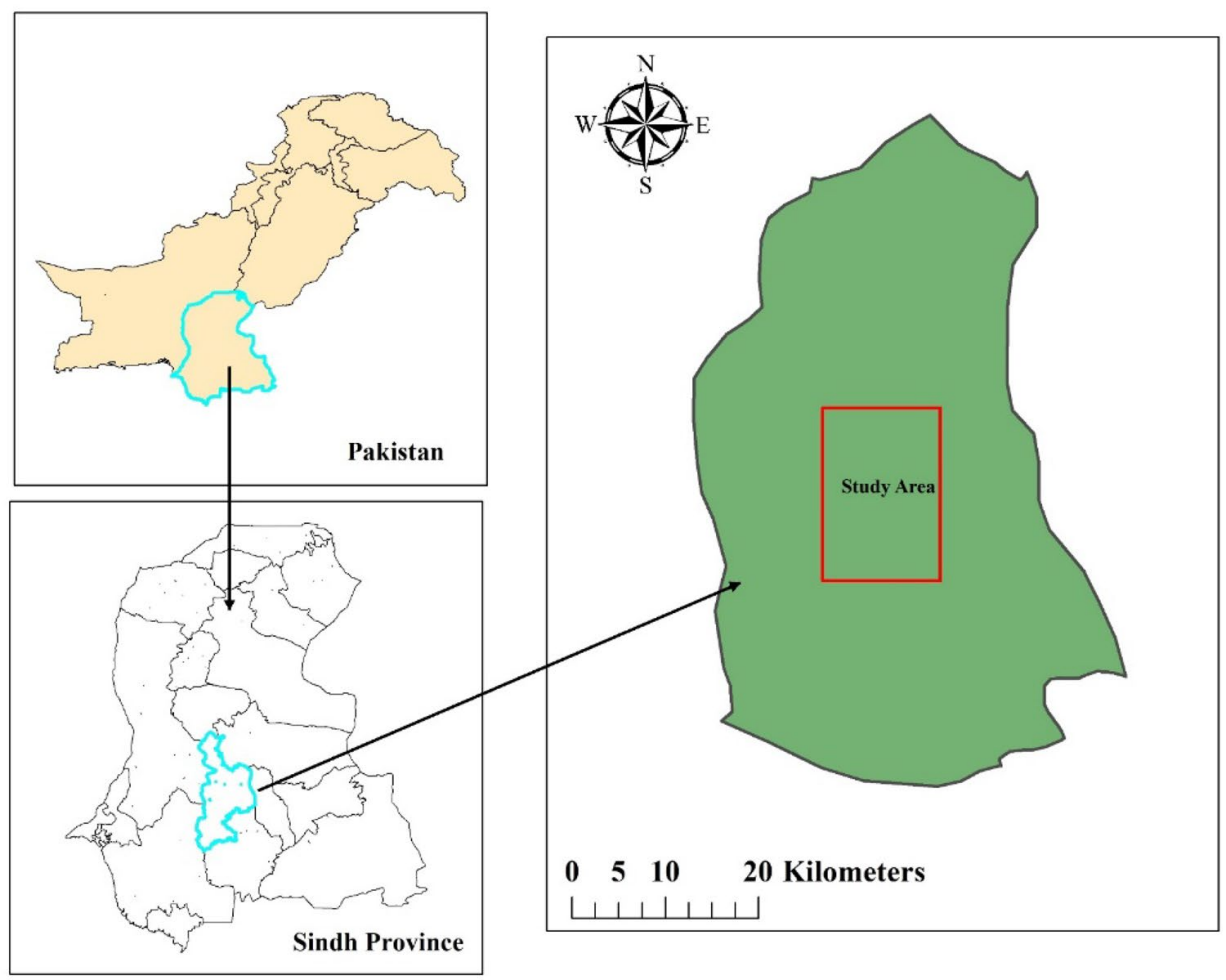
Location map of study area.

The fertilizer sources used in the experiment were superphosphate (P_2_O_5_, 12%) and potassium chloride (K_2_O, 60%). Treatments fallow as T0 (no fertilizer was applied (control), T1 (applied P at a level of 90 kg ha^−1^), T2 (applied K at the level of 90 kg ha^−1^), and T3 (applied both PK together at the level of 90 kg ha^−1^), respectively. The leguminous species seed was soaked in water for one day before sowing. After the preparation of soil for the pot experiment, ten seeds were sown in each pot and covered with dry soil. After plant development, five vigorous plants in every pot allowed to be examined were selected. While distilled water was commonly used for irrigation purposes. Leguminous species Chickpeas (*Cicer arietinum* L) and Peas (*Cicer arietinum* L), fallowed as cultivars (NIAB Channa-2016) and (Sarsabz) were seeded on 1st November 2020. Figure 1 shows the geographical location of the surveyed region.

### Plant sampling and analysis

At the full bloom stage, on 5th March 2021 both leguminous species were harvested, and the plant shoot samples were separated from the pots. After that, plant root samples were removed from the pots and washed thoroughly to remove any remaining soil. Leguminous fresh shoot and roots biomass samples were weighed, dried for 72 h at 65 °C in an oven, crushed, and stored for nutrient analyses. Samples of plant shoots and root were burned at high temperatures to determine the N, P, and K contents using a solution of sulphuric acid (H_2_SO_4_) and hydrogen peroxide (H_2_O_2_) diluted. Further, a previously described Kjeldahl digestion method, was used to assess the amount of N in plant shoot and root biomass; the molybdovanadate method was used to determine the amount of P in plants^41^, while determination of K content in plants flame photometer was used to analysis^42^.

### Soil determination

Following plant harvesting, soil samples were collected from each pot, packed, and then transported to the laboratory for analysis. After that, the samples were separated into two main parts, and immediately kept one portion at − 4 °C for soil moisture content, inorganic nitrogen, and enzyme analysis.

The second portion of samples was air-dried and passed through a 2-mm sieve for the determination of pH range, available P, K contents. Additionally, we passed the sub-samples through a 0.25-mm sieve to examine soil total nitrogen (TN) and SOM. Analysis of soil inorganic nitrogen contents by the Kjeldhal method of steam distillation, as described in an earlier study^43^. Oven drying at 105 °C for 48 h determined soil moisture. Soil TN content was examined by the Kjeldhal method described in previous research^44^. An earlier Walkley–Black method, was used for the determination of SOM^44^. Soil electrical conductivity (EC) and pH were determined by preparing a ratio of 1:2.5 soil/water in order to use an EC meter (Hanna Model-8733, Germany) and a pH meter (Jenway, Model-3510, Gransmore Green, Felsted, Dunmow, Essex, CM6 3LB, UK), respectively. According to^45,46^, microbial biomass phosphorus (MBP) was examined in fresh, wet soil using a chloroform-fumigation extraction method. To determine the total P in the soil, samples are digested in a solution of perchloric acid (HClO_4_) and nitric acid (HNO_3_) in a ratio of 1:3. According to the procedure described in^47^, a 0.5 M NaHCO_3_ extract solution was used to determine soil Olsen P. The contents of total P and available P were analyzed using a spectrophotometer with visible blue light (Model UV-2100, Shimadzu, Tokyo, Japan). For available K content, 5 g of dry soil was extracted with one molarity of NH4OAc and determined on a flame photometer^48^.

### Soil enzyme activities

All enzyme activities were determined in fresh soil samples collected after harvesting the legume species. Before the enzyme activity assay, a portion of the subsamples were oven dried at 105 °C for 24 h to determine soil moisture. The activities of the urease enzyme were investigated by the previously described colorimetric method^49^. Firstly, 5 g of fresh soil was incubated for 2 h at 36 °C, and urea solution was used as a substrate. An altered Berthelot reaction was used to release NH_4_^+^ through the potassium chloride (KCl) solution. The β-glucosidase activity was assessed in 1 g of moist soil (< 2 mm) kept in a tube, treated with 0.25 ml of toluene, and 4 ml of adjusted universal buffer (pH 6), added 1 ml of PNG solution (25 mM) and placed in an incubator for 1 h at 37 °C^50^. After incubation, 1 ml of CaCl_2_ solution and 4 ml of Tris buffer (pH 12) were added, and absorbance at 400 nm was measured using a spectrophotometer (RIGOL, USA). As described by^51^. Phosphatase activity has been investigated using the substrate p-nitrophenyl phosphate (r-NPP). After that, 5 g of wet soil was mixed with 20 ml of acetate buffer (pH 5.2) and 100 mMof r-NPP and kept for 30 min in an incubator at 30 °C. To determinate the reaction, 1 ml of CaCl2 and 4 ml of 0.2 MNaOH were added after incubation. The absorbance was measured with a spectrophotometer set to 405 nm (RIGOL, United States). The p-nitrophenol produced method after the soil was incubated with added substrate and acetic buffer (pH 5.5) for an hour at 37 °C was used to evaluate the N-acetyl-D-glucosaminidase enzyme. At 400 nm, the filtrate's intensity of its yellow colour was measured^52^. The activities of enzyme units have been calculated in Table 1.

**Table 1:** Present the various enzymes composition and enzyme commission quantity (EC), corresponding substrate, and enzyme units.

Enzymes	Unit	Substrate	EC
Urease	µg N g^−1^ soil 2 h^−1^	Urea	3.5.1.5
β-glucosidase	μg PNG g^−1^ dwt h^-1^	p-nitrophenyl phosphate	3.2.1.21
Phosphatase	μg p-NPP g^−1^ h^−1^	p-nitrophenyl phosphate	3.1.3.1
N-acetyl-glucosaminidase	mmol pNP kg^−1^ h^−1^	p-nitrophenyl-N-acetyl-β-D-glucosaminide	3.2.1.30

### Statistical analyses

IBM SPSS Statistics version 20.0 (Corp., Armonk, NY, USA) was used to evaluate a one-way analysis of variance (ANOVA) to see if there were any significant effects of fertilizer treatments on legume nutrient accumulation. Investigate the fertilizer application by using Tukey’s multiple variable levels (*p* < 0.05). The significant correlation pattern of the study has been examined using IBM SPSS by applying Pearson’s correlation and indicating the significant range at ** *p* < 0.01 and * *p* < 0.05. Origin software, using Origin Pro 9.0, created the graphs for the enzymatic activity determination. The principal component analysis (PCA) was performed to determine the general relationship between various cultivars, enzymatic activities, and soil characteristics using CONOCO (version 5) at a *p* < 0.05 level.

### Ethical approval and consent to participate

This study does not include human or animal subjects.

## Results

### Shoot biomass yield

Application of fertilizers has significant effects on chickpea and pea shoot dry biomass yield and root biomass (Table 2). Chickpeas presented the highest dry shoot biomass yield in T3 treatment by 33.8% as compared with T0 treatment. A lower amount of biomass yield increased in T1 treatments by 12.0% compared to T0 treatments. The T3 treatment recorded the highest root biomass of chickpeas at 21.2%, while the T2 treatment recorded the lowest root biomass at 12.1%, compared to the control. However, chickpea species decreased root biomass in T1 by 48.1% compared to the control. In the case of pea species, the maximum shoot dry biomass yield in the T1 treatment increased by 19.9% compared to the control. Despite that, T3 treatment also increased the dry biomass yield of pea species by 15.3%, higher than control. Root dry biomass and nutrient uptake capability of pea species showed significant changes under fertilization treatments. The pea species root dry biomass increased in the following pattern: T3, and T2, by 148.9% and 65.9%, respectively, compared to the T0 treatment.

**Table 2:** Effects of fertilizer treatments on shoot biomass yield and root biomass g pot^-1^ of chickpea and pea (repeats* n* = 4, average ± standard deviation).

Species	Chickpea		Pea	
Parameters	Shoot dry biomass yield (g pot^−1^)	Root dry biomass yield (g pot^−1^)	Shoot dry biomass yield (g pot^−1^)	Root dry biomass yield (g pot^−1^)
T0	30.19 ± 1.02b	4.052 ± 1.066a	20.14 ± 1.599a	1.73 ± 0.572c
T1	33.06 ± 3.74ab	2.082 ± 0.649b	24.15 ± 5.815a	1.37 ± 0.453bc
T2	39.72 ± 6.70a	4.390 ± 1.335a	19.25 ± 2.136a	2.880 ± 0.503b
T3	40.42 ± 3.65a	4.935 ± 0.896a	23.29 ± 1.690a	4.320 ± 0.760a

Small letters show a significant difference at *P* < 0.05, based on Tukey’s multiple test.

*Note*: T0 (no fertilizer was applied), T1 (applied P at a level of 90 kg ha^−1^) and T2 (applied K at a rate of 90 kg ha^−1^) and T3 (applied both P and K at the level of 90 kg ha^−1^): P (P_2_O_5_) and K (K_2_O) fertilizers.

### Chickpea shoot nutrient uptake

Significant changes were observed in shoot nutrient uptake during different fertilizer treatments, as presented in Table 3. However, T1 treatment promoted the N uptake ability by 37.0%, compared to the control. While the minimum shoot N uptake (11.0%) was noted in T2 treatment compared to the T0. Whereas P and K uptake of chickpeas increased significantly in T2, i.e., 52.2% and 44.2%, respectively, compared to the control. The least shoot P uptake in chickpea was noted in T1 by 18.1% compared to the control. Shoot K uptake of chickpea was decreased in T3 treatment, which was 7.7% compared to T0.

**Table 3:** Effects of fertilizer treatments on shoot N, P and K uptake g pot^−1^ of chickpea species (repeats* n* = 4, average ± standard deviation).

Species	Chickpea		
Parameters	Shoot N uptake (g pot^−1^)	Shoot P uptake (g pot^−1^)	Shoot K uptake (g pot^−1^)
T0	0.730 ± 0.101a	0.103 ± 0.027a	0.858 ± 0.137b
T1	1.00 ± 0.253a	0.122 ± 0.028a	0.870 ± 0.096b
T2	0.816 ± 0.134a	0.157 ± 0.054a	1.205 ± 0.202a
T3	0.983 ± 0.097a	0.142 ± 0.041a	0.792 ± 0.089b

Small letters show a significant difference at *P* < 0.05, based on Tukey’s multiple test.

*Note*: T0 (no fertilizer was applied), T1 (applied P at a level of 90 kg ha^−1^) and T2 (applied K at a rate of 90 kg ha^−1^) and T3 (applied both P and K at the level of 90 kg ha^−1^): P (P_2_O_5_) and K (K_2_O) fertilizers.

### Chickpea root nutrient uptake

Table 4 showed the variation in root nutrient (N, P, and K) uptake ability of chickpea species during fertilization. Therefore, compared to the control, chickpea species reduced their N uptake abilities under fertilizer treatments. In contrast, fertilizers treatment enhanced root P and K uptake, the maximum P and K uptake of root was recorded in the T3 treatment, i.e., 26.2 and 13.8%, respectively, as compare with T0 treatment. The minimum root P and K uptakes of chickpea cultivars were found in T2 at 15.6% and 8.9%, respectively, compared to no fertilizer applied.

**Table 4:** Effects of fertilizer treatments on root N, P and K uptakes g pot^−1^ in chickpea species (repeats* n* = 4, average ± standard deviation).

Species	Chickpea		
Parameters	Root N uptake (g pot^−1^)	Root P uptake (g pot^−1^)	Root K uptake (g pot^−1^)
T0	0.134 ± 0.25a	0.019 ± 0.005a	0.102 ± 0.03a
T1	0.096 ± 0.43ab	0.013 ± 0.003a	0.051 ± 0.003b
T2	0.059 ± 0.20ab	0.022 ± 0.008a	0.111 ± 0.005a
T3	0.089 ± 0.21ab	0.024 ± 0.003a	0.116 ± 0.002a

Different small letters show a significant difference at *P* < 0.05, based on Tukey’s multiple test.

*Note*: T0 (no fertilizer was applied), T1 (applied P at a level of 90 kg ha^−1^) and T2 (applied K at a rate of 90 kg ha^−1^) and T3 (applied both P and K at the level of 90 kg ha^−1^): P (P_2_O_5_) and K (K_2_O) fertilizers.

### Pea shoot nutrient uptake

Fertilizer treatments have significant effects on nutrients such as N, P, and K uptake in pea shoots as presented in (Table 5). The greater N uptake ability was observed in T3 by 151.4% compared to the control. The lowest N uptake was recorded for T1 treatment by 6.5% compared to the control. On the other hand, pea species had a high amount of P uptake in their shoot under T3 (49.2%), and the minimum shoot P uptake was detected in the T1 by 27.2% compared to the T0 treatment. The maximum K uptake ability was noted in T1 (50.3%), compared to the control. While shoot K uptake was reduced in T3 treatment in comparison with control.

**Table 5:** Effects of PK fertilizer treatments on shoot N, P, K uptake g pot^-1^ of pea species (repeats* n* = 4, average ± standard deviation).

Species	Peas		
Parameters	Shoot N uptake (g pot^−1^)	Shoot P uptake (g pot^−1^)	Shoot K uptake (g pot^−1^)
T0	0.505 ± 0.037a	0.061 ± 0.014b	0.801 ± 0.173b
T1	0.538 ± 0.121a	0.078 ± 0.018ab	1.204 ± 0.202a
T2	0.469 ± 0.60a	0.087 ± 0.015ab	0.870 ± 0.096b
T3	0.556 ± 0.45a	0.091 ± 0.013a	0.792 ± 0.089b

Different small letters show a significant difference at *P* < 0.05, based on Tukey’s multiple test.

*Note*: T0 (no fertilizer was applied), T1 (applied P at a level of 90 kg ha^−1^) and T2 (applied K at a rate of 90 kg ha^−1^) and T3 (applied both P and K at the level of 90 kg ha^−1^), P (P_2_O_5_) and K (K_2_O) fertilizers.

### Pea root nutrient uptake

Significant modifications were recorded in the nutrient uptake of pea species roots during fertilizer treatments (see Table 6). The maximum root N uptake increased in T3 by 151.4%, while root N uptake decreased by 32.3% in T1 compared to T0 treatment. In comparison to the control, the T3 treatment increased root P uptake by 100.1%, and the lower root P uptake was 40.0% observed in the T1 treatment. However, the greater root K uptake of pea species was noted in the T3 treatment by 13.0%, and the root K uptake was reduced in the T1 treatment by 3.1% compared to the T0 treatment.

**Table 6:** Impact of PK fertilizer treatments on root N, P, and K uptake (g pot^-1^) in pea species (repeats* n* = 4, average ± standard deviation).

Species	Peas		
Parameters	Root N uptake (g pot^−1^)	Root P uptake (g pot^−1^)	Root K uptake (g pot^−1^)
T0	0.044 ± 0.022b	0.010 ± 0.001a	0.107 ± 0.003b
T1	0.030 ± 0.010b	0.014 ± 0.001a	0.103 ± 0.001b
T2	0.058 ± 0.008b	0.019 ± 0.001a	0.106 ± 0.001b
T3	0.110 ± 0.036a	0.020 ± 0.001a	0.121 ± 0.002a

Different small letters show a significant difference at *P* < 0.05, based on Tukey’s multiple test.

*Note*: T0 (no fertilizer was applied), T1 (applied P at a level of 90 kg ha^−1^) and T2 (applied K at a rate of 90 kg ha^−1^) and T3 (applied both P and K at the level of 90 kg ha^−1^), P (P_2_O_5_) and K (K_2_O) fertilizers.

### Soil nitrogen contents

The soil mineral nitrogen content affected by P and K fertilizer treatments during the planation of legume species is shown in Table 7. The T2 treatment increased the NH_4_^+^ and NO_3_^-^ content, i.e., 15.3% and 16.6% in chickpea species respectively, higher than control. While the T3 treatment reduced the NH_4_^+^ content by 2% and T1 decreased the NO_3_^−^ content by 15% in chickpeas as compared to the T0 treatment. In the case of pea species, the T2 improved the NH_4_^+^ and NO_3_^−^ content of the soil, i.e., 21.1% and 34.4%, respectively, compared to the control.

**Table 7:** Influences of fertilizer treatments on soil mineral nitrogen NH_4_^+^ and NO_3_^**-**^ (mg kg^−1^) (repeats* n* = 4, average ± standard deviation).

Species	Chickpea		Pea	
Treatments	NH_4_^+^ (mg kg^−1^)	NO_3_^-^ (mg kg^−1^)	NH_4_^+^ (mg kg^−1^)	NO_3_^−^ (mg kg^−1^)
T0	4.32 ± 0.55a	24.7 ± 3.40ab	4.32 ± 0.55a	8.23 ± 1.28bc
T1	4.47 ± 0.44a	20.9 ± 3.19b	4.97 ± 0.56a	6.91 ± 1.41c
T2	4.98 ± 0.44a	28.8 ± 2.14a	5.23 ± 0.51a	11.07 ± 0.64a
T3	4.20 ± 0.14a	25.5 ± 2.61ab	4.30 ± 0.14b	10.02 ± 1.06ab

Different small letters show a significant difference at *P* < 0.05, based on Tukey’s multiple test.

*Note*: T0 (no fertilizer was applied), T1 (applied P at a level of 90 kg ha^−1^) and T2 (applied K at a rate of 90 kg ha^−1^) and T3 (applied both P and K at the level of 90 kg ha^−1^), P (P_2_O_5_) and K (K_2_O) fertilizers.

### Soil MBP and available P and K contents

Soil microbial biomass phosphorus (MBP) and available P and K contents of soil changed with fertilizer treatments when leguminous species were grown (Table 8). The percentages of MBP in T3 (95.7%), T1 (90.3%), and T2 (79.2%) in chickpea species were greater than control. The Olsen P of chickpea species was increased in T2 and T3, i.e., 36.3% and 28.9%, respectively, compared to T0 treatments. While the P content of soil was decreased in T1 treatment by 32.9% compared to control. Soil available K content showed an increasing trend in the following directions: 16.7%, 32.8%, and 42.9% for T1, T2, and T3, respectively, compared to T0 treatment. In the case of pea species, T3 treatment increased the highest range of MBP by 81.5%, and the T1 recorded the lowest MBP at 9.31%, compared to the T0 treatment. The maximum soil available P content observed in T2 was 34.8%, and T3 also improved P content by 31.0% as compared to T treatment. The greater K content of soil was in T3 by 28.6%, when compared with control. Soil available K content decreased in T1 by 5.2% compared to control.

**Table 8:** Influence of fertilizer treatment on MBP, available P and K contents of soil (repeats* n* = 4, average ± standard deviation).

Species	Chickpeas			Peas		
Treatments	MBP(mg/kg)	AP (mg/kg)	AK(mg/kg)	MBP (mg/kg)	AP (mg/kg)	AK (mg/kg)
T0	48.21 ± 2.0b	22.05 ± 0.43b	107.7 ± 4.29c	49.36 ± 7.223b	16.85 ± 1.666b	92.4 ± 8.668b
T1	91.75 ± 3.542a	14.78 ± 2.42c	125.9 ± 11.8b	51.29 ± 6.157b	20.40 ± 0.122a	87.8 ± 4.487b
T2	86.42 ± 3.725a	30.08 ± 1.592a	145.2 ± 10.2ab	68.47 ± 3.53ab	22.72 ± 1.066a	92.9 ± 6.285b
T3	94.39 ± 1.606a	28.43 ± 1.23a	153.7 ± 5.80a	89.62 ± 3.320a	22.07 ± 0.554a	119.2 ± 9.24a

Different small letters show a significant difference at *P* < 0.05, based on Tukey’s multiple test.

*Note*: T0 (no fertilizer was applied), T1 (applied P at a level of 90 kg ha^−1^) and T2 (applied K at a rate of 90 kg ha^−1^) and T3 (applied both P and K at the level of 90 kg ha^−1^), P (P_2_O_5_) and K (K_2_O) fertilizers.

### Influences of fertilization treatments on Enzymes activities

**Figure 2:**
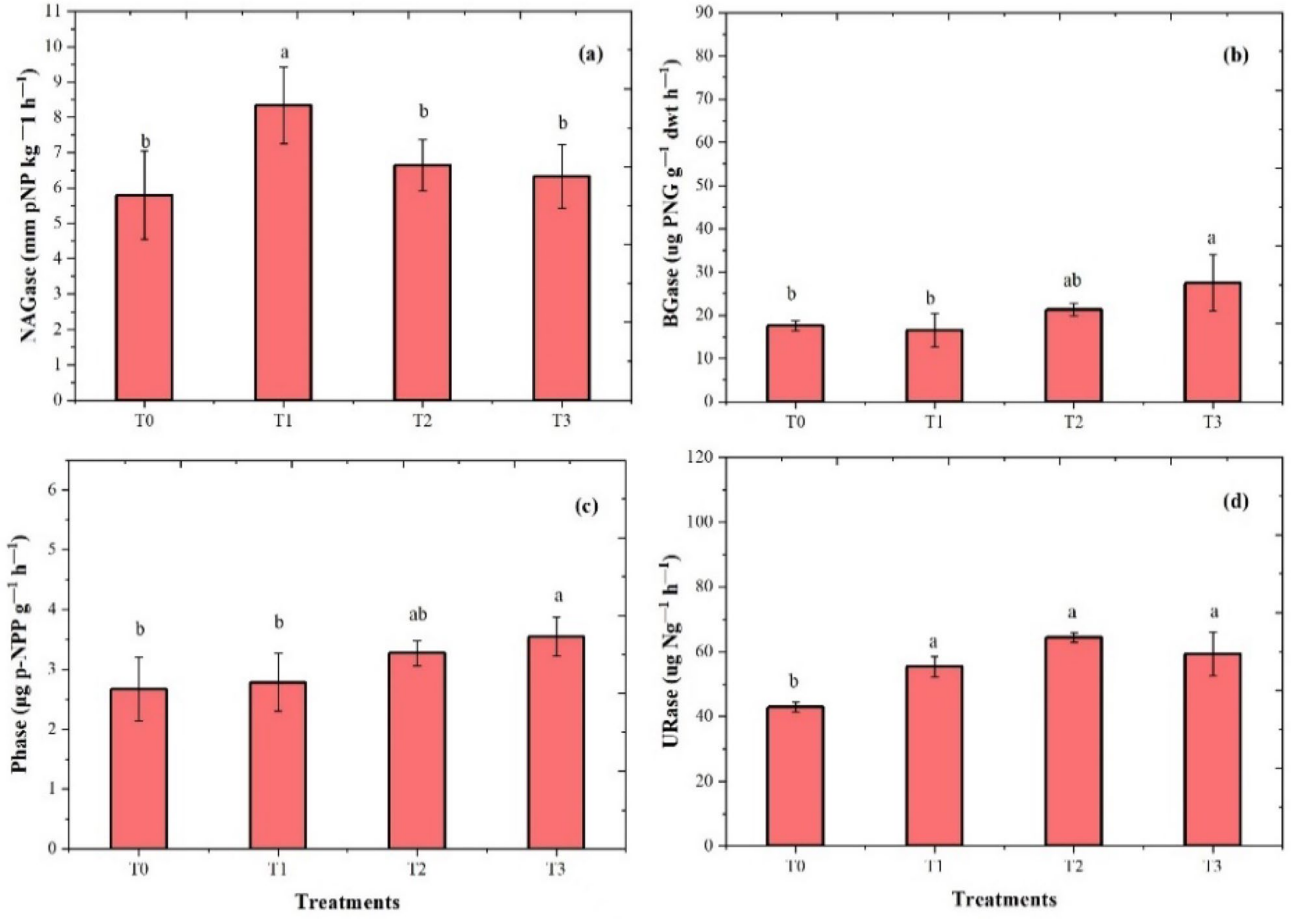
Effects of fertilization on soil enzyme activities after harvesting the chickpea species: (**a**) N-acetylglucosaminidase (NAGase), (**b**) β-glucosidase (BGase), (**c**) phosphatase (Phase), and (**d**) urease (URase). Bars with different small letters indicate significant variation at the (*p* < 0.05) level based on Tukey’s multiple test (repeats *n* = 4).

Fertilizer treatments showed a significant effect on soil enzymatic (i.e., N-acetyl-β-D-glucosaminidase (NAGase), β-glucosidase (BGase), phosphates (Phase), and urease URase) activities after harvesting the chickpea species, as shown in Fig. 2. T1 showed the highest NAG activity at 52.4%, while T2 and T3 also increased, i.e., 21.6% and 15.5% activity, respectively, in chickpeas compared to T0. However, both BGase and Phases enzyme activities were higher in T3, i.e., 55.8% and 33.9%, respectively, as compared to the control. While T2 treatment improved soil URase enzyme activities by 50.1% compared to the control.

**Figure 3:**
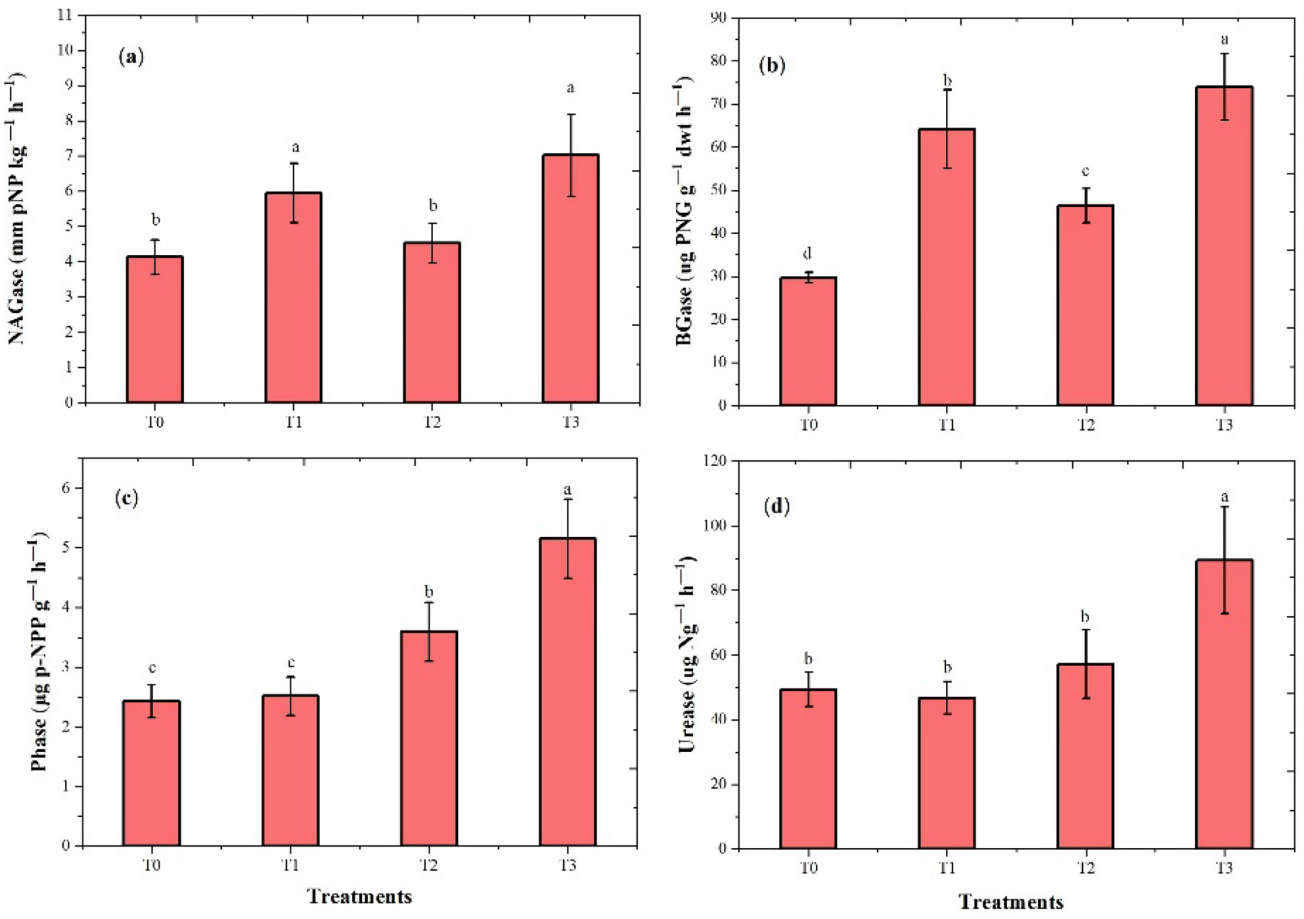
Impact of fertilizer treatments on soil enzyme activities after harvesting the pea species: (**a**) N-acetylglucosaminidase (NAG), (**b**) β-glucosidase (BG), (**c**), phosphatase (Phase), and (**d**) urease (Urease). Bars with different small letters indicate significant variation at the (*p* < 0.05) level based on Tukey’s multiple test (repeats *n* = 4).

All fertilizer treatments enhanced the activity of extracellular enzymes in soil, such as NAGase, BGase, Phase, and URase after the harvesting of pea species (Fig. 3). Maximum NAGase activities were found in T3 treatments (70.0%), and the lowest activity recorded in T2 was 9.6% as compared to the T0 treatment. The T3 treatment significantly increased BGase enzyme activities by 149.7% compared to the control. However, the maximum Phase and URease activities were also prominent in T3, i.e., 81.1% and 111.9%, respectively, which is higher than in T0 treatment.

### Correlation between soil enzymes and soil properties

However, analysis of Pearson’s correlation (r) shows soil enzymes NAGase, BGase, Phase, and URase activities are significantly correlated with soil properties after harvesting the chickpea species during fertilization (Table 9). The NAG was significantly positively correlated with SOM and MBP (r = 0.502* and 0.543*, respectively). While the BG enzyme found a significant positive correlation with TN and AK (r = 0.517*, and 0.613*).The Phase activities indicated a strong and significant relationship with MBP and AK (r = 0.508*, 0.648**). However, the URase activities, also significantly related to MBP (r = 0.770**) and AK (r = 0.779**).

**Table 9:** Relationship between soil enzymes and soil properties during chickpea planting under fertilization treatment.

Parameters	SOM	TN	NH_4_^+^	NO_3_–	MBP	AP	AK	pH
NAGase	0.502*	− 0.566*	0.208	− 0.440	0.543*	− 0.458	0.089	0.143
BGase	0.359	0.517*	− 0.197	0.239	0.354	0.464	0.613*	− 0.399
Phase	0.471	0.317	0.316	0.240	0.508*	0.380	0.648**	0.182
Urease	0.265	0.179	0.280	0.099	0.770**	0.412	0.779**	− 0.142
SOM	1	0.321	0.091	0.534*	− 0.027	0.536*	0.356	0.055
TN		1	0.302	− 0.036	0.011	0.315	0.075	0.090
NH_4_^+^			1	0.021	0.128	0.527*	0.269	− 0.110
NO_3_^−^				1	0.032	0.005	0.715**	0.192
MBP					1	0.542	0.608	− 0.211
AP						1	0.876	0.402
AK							1	0.321
pH								1

*Note* NAGase (N-acetylglucosaminidase), BGase (β-glucosidase), Phase (phosphatase), and URase (urease) are significantly related to soil properties. OM (organic matter), TN (total nitrogen), NH_4_^+^ (ammonia) and NO_3_^−^ (nitrite), AP and AK (available phosphorus and potassium), and pH (soil pH), the * indicate the significant range at *p* < 0.05, and ** showed significant level at *p* < 0.01.

Based on Pearson’s correlation (r) analysis, the soil enzymes such as NAGase, BGase, Phase, and URase activities are considerably correlated with soil properties once harvested the pea species under PK fertilization (Table 10). The NAG enzyme was significantly positively related to SOM and K content (r = 0.505* and 0.611**, respectively), and BG showed a significant negative interaction only with soil TN (r = 0.517*). However, phase enzymes showed a significant negative relationship with MBP and AK, r = − 0.831** and − 0.822**, respectively. In contrast, a positive relationship is presented with AP r = 0.498*. Urease is significantly positively related to MBP and AP (r = 0.743**, 0.498*), and a negative relationship is seen with available K (r = − 0.686**). Non-significant associations were observed between soil enzymatic activities and other soil properties.

**Table 10:** Relationship between enzymes and soil properties during pea planting under PK fertilization.

Parameters	SOM	TN	NH_4_^+^	NO_3_^-^	MBP	AP	AK	pH
NAGase	0.505*	0.371	0.356	− 0.120	0.414	0.398	0.611*	0.202
BGase	0.354	− 0.511*	0.251	− 0.004	0.485	0.483	0.454	0.145
Phase	0.295	0.143	0.063	− 0.305	− 0.831**	0.498*	− 0.822**	0.041
URase	0.401*	− 0.269	0.137	− 0.185	0.743**	0.498*	− 0.686**	− 0.233
SOM	1	− 0.102	− 0.149	− 0.225	0.290	0.118	− 0.146	0.478
TN		1	0.016	0.132*	− 0.131	0.136	0.135	− 0.047
NH_4_^+^			1	0.432	− 0.133	0.436	− 0.201	0.150
NO_3_^−^				1	− 0.573*	0.381	0.216	− 0.123
MBP					1	0.479	1.608*	0.265
AP						1	0.393	− 0.304
AK							1	− 0.120
pH								1

*Note* NAGase (N-acetylglucosaminidase), BGase (β-glucosidase), Phase (phosphatase), and URase (urease) are significantly related to soil properties: SOM (soil organic matter), TN (total nitrogen), NH_4_^+^ (ammonia) and NO_3_^−^ (nitrite), AP and AK (available phosphorus and potassium), and pH (soil pH), the * and ** indicates the significant range at *p* < 0.05, and at *p* < 0.01.

### Correlation among legume nutrient accumulation, soil properties, and enzymatic activities

**Figure 4:**
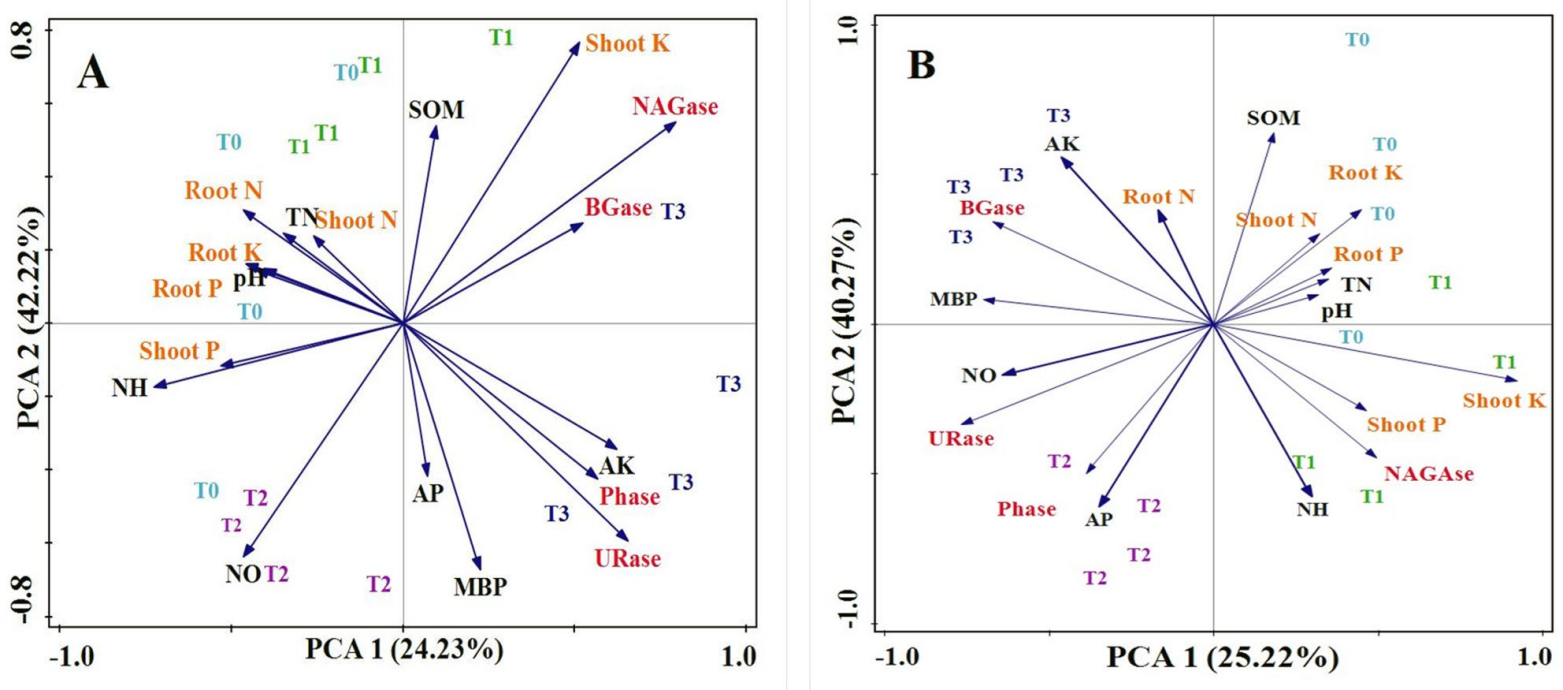
PCA analysis showed an overall relationship between shoot and root PK uptake of plants, soil enzymes, and soil properties in two different legumes: (**A**) Chickpeas and (**B**) Peas. The position of variables showed relationships to each other. *Note* NAGase (N-acetylglucosaminidase), BGase (β-glucosidase), Phase (phosphatase), URase (urease) properties, TN (total nitrogen), NH_4_^+^ (ammonia) and NO_3_^−^ (nitrate) SOM (soil organic matter), and AP, AK (available phosphorus and potassium).

Based on principle component analyses, an overall correlation was seen among shoot and root PK uptake, soil properties, and enzymatic activities under PK fertilizer treatments (Fig. 4). During plantation chickpea cultivation, the first axis attributed 24.23% and the second axis 42.2% of the changes in plant PK uptake abilities, soil properties, and soil enzymes for chickpea species. T1 and T0 treatments clustered with chickpea root N, P, and uptakes, soil TN and soil pH. Soil characteristics such as available P and K content, MBP, and soil enzymes, Phase and URase presented a significant relationship followed by T3 treatment. While T2 cluster with shoot P uptake and NH_4_^+^and NO_3_–N.., The first and second axes contributed 25.2% and 46.2%, respectively. An opposite trend was noted among treatments, soil enzymes, and properties for pea species The results of the PCA analysis showed various fertilizer treatments presented interactions with PK uptake, soil properties, and soil enzymes. The T2 treatment linked soil properties (NO_3_ − , available P) and soil enzymes (phase and urease). Soil available K content, MBP and BGase enzymes, and root N uptake were all grouped together in the T3 treatments. While T0 and T1 treatments clustered with shoot and root N, P, and K uptake and soil properties, i.e., SOM, pH, TN, and NH_4_^+^., only NAGase soil enzymes were related to the T0 treatment.

## Discussion

### Effects of fertilizer on plant nutrient abilities

This study investigated the shoot and root biomass and phosphorus and potassium uptake abilities of two leguminous species, such as chickpea and pea species. The accumulation of biomass yield and the ability to acquire nutrients such as N, P, and K from the soil depend on fertilization techniques. However, in this study, fertilizer treatments increased biomass production and N, P, and K uptake abilities in both legumes. The PK fertilizer effects of N-uptake legumes are famous for their root nodule nitrogen fixation ability and nitrogen transfer capacity to the soil, which decrease competition with crops for nutrient uptake^53^. There is an extensive amount of researches are present about the quantity of nitrogen that legumes transfer to related crops. It fluctuates according to agronomic parameters like weather patterns and symbiotic effectiveness as well as conditions that affect legume N-fixation, such as species of legumes. In general, because the soil’s available nutrients change rapidly in the soil, they will be affected by many factors, including farming methods, fertilizer types, fertilizer application methods, and crop growth^54^. The previous finding showed that fertilizer input can improve plant biomass yield and is essential for enhancing agronomic efficiency, while a higher rate of fertilizer can have an adverse effect on plant growth^55^. Studies from the past showed that the levels of P and K in soils that had been treated with fertilizer were much higher. Another study^56^ demonstrated that legumes take up K from K-fertilized soils^57^. Phosphorus and K fertilization can improve nutrient uptake for legume crop growth. Plants with higher P and K uptake from the soil develop utilization in processes that promote quicker development and supply higher nutrient percentages to the above-ground plant parts^58^. Similarities were seen in other studies; for example, the P fertilizer is a parameter that reflects the plant nutrient (P) uptake capacity from the rhizosphere soil^59^. The excess P may accumulate in the inorganic and organic reservoirs, resulting in highly saturated PO_4_^3^^−^ pools in the soil. Phosphorus uptake does not depend on the present soil P content but is relative to the available (Olsen) P that plants may use^60^. The uptake of nutrients such as P and K during plant growth and biomass production depends on the root length, density, and root depth of a plant during its growing period^61^.

### Impact of fertilization on soil nutrients

Our results showed significant differences in soil MBP under fertilization treatments. Generally, variations in soil MBP may depend on the soil amendment. Previous research supports the current study; results showed that oats were grown as green manure with chemical fertilizer applications such as N, P, and K and without organic fertilizer. The soil biomass P might have improved without the incorporation of organic fertilizer. This may happen because some limiting factors can help to easily decompose accessible P in soil, which was required to enhance biomass P in the soil. This development of microbial biomass P could increase when only inorganic fertilizer was applied^62^. During the P fertilization, when plant roots and microbes interacted with each other, microorganisms could release organic acids, phosphatases, enzymes, and other substances to stimulate soil insoluble phosphorus in the form of microbial biomass phosphorus, thereby improving soil phosphorus bioavailability^46^. In the present study, fertilization strategies could improve soil P and K contents. Similarly, it has been demonstrated that soil available P and K levels increased after receiving PK fertilization and were higher than in the non-P and K applied plots and other treatments^57^. Multiple studies have proposed that chemical fertilizer can improve soil physical and chemical properties as well as increase soil Olsen-P. Because the soil’s available nutrients change quickly in the soil, they will be affected by many factors, including farming methods, fertilizer types, fertilizer methods, and plant development^37,63^.

The current study showed T2 treatments where K fertilizer applied were more effective for soil P content. However,^34^ say that increasing the amount of P in the soil may involve a number of reactions, such as the pH of the soil, its sorption capacity, and the root exudation of legume species. Also, the properties of the soil affect how much P is available. In calcareous soils, P treatment often makes P less available because Ca-P minerals form and stick to the soil. The immobilized P will be released slowly; therefore, the released P comes from physico-chemical adsorption reactions. If the immobilized phosphorus is passed from one generation of microbes to the next, then there will be constant competition between microbes and plants for phosphorus^64^. This implies that the slow release of immobilized phosphorus contributes to the soil's available phosphorus. Measuring soil characteristics is crucial when fertilizer is extremely concentrated, as P and K fertilizer sources play a significant role When fertilizer is used, the K level in the soil goes up because some of the K in the fertilizer is fixed as non-exchangeable K and then slowly released to the soil in the form of available K content^65^.

### Soil extracellular enzymatic activities

Plants and microbes can produce enzymes, which play an extensive role in increasing macronutrients in the soil^66^. Our results have indicated that application of P and K fertilizer affect enzyme activities during the cultivation of legume crops (Figs. 2, 3). The previous finding supports the current study, which demonstrates that the activities of enzymes showed increasing trends in response to applied chemical fertilization in soil^67^. The (T1) P fertilizer increased NAGase in chickpea species, while theT3 treatment improved NAGase activity in pea species. The NAGase enzyme activity is important for plant development and plays an important part in the N cycle, and the breakdown of amino acids into sugars can mineralize N in soil. On the other hand, excessive amounts of chemical fertilizer decreased soil enzyme activities^68^.

This study found that BGase and Phase activities increased when fertilizer was applied. According to an earlier study, BGase activities developed with inorganic fertilization treatment are commonly known as a sensitive factor in soil fertility and quality^69,70^. The PK addition resulted in a considerable increase in soil respiration which suggests that higher microbial activity in the presence of addition P allows for faster transformation of soil organic matter. Previous research indicates improved acid phosphate (AP) activity in the soils under fertilizer application^71^. Phosphatase activities are a key indicator of the soil's P cycle, and more activity can lead to a rise in the number of microorganisms and change the soil's properties^30^. The resulting P and K addition could proliferate the required P in the soil and restrict the availability of P, which can increase and maximize soil microbes’ activity. Soil microorganisms capable of secretion release large amounts of phosphorus activity into the soil^31^. Additionally, the results of the experiments showed that P and K treatments upgraded maximum urease activity. Different soil characteristics, such as soil pH, nutrient availability in the soil, and fertilizers, have an impact on urease enzyme activity^72^. Urease can regulate N availability for plant growth and could hydrolyze urea into ammonia and CO_2_^73^.

### Interaction between plant nutrient enzymatic activities and soil properties

The current research examines the enzymatic activities of soil, which can have integrated effects on soil properties under PK fertilizer treatments. These results could support a part of the current study's hypothesis that soil enzymes and soil microbial biomass P can increase nutrient availability. The Pearson correlation analysis showed significant relationships between enzyme activities, and soil properties under fertilization when two legumes were planted. However, PCA analysis showed an overall relationship between legume nutrient uptake and soil characteristics, MBP, and soil enzymes. While PCA analysis indicated a 24% and 25% total modification in the uptake of N, P and K, enzymes, and soil properties for both species after fertilization. The PCA graph showed that there was an interaction between nutrient uptake and MBP. Although there has been competition between plants and microbes, the presence of increases in soil microbial biomass phosphorus (MBP) could enhance the uptake of phosphorus by plants^74^. The MBP pool provides an important supply of available phosphorus (P) in soils. Even when Olsen P levels are high, the MBP pool continues to provide an adequate quantity of inorganic P, which plants can utilize more efficiently. However, the NAGase and urease N-related enzymes are positively related to SOM and MBP while also showing a significant negative interaction with TN during legume cultivation. Similarly, an earlier study demonstrates that legume species, such as alfalfa, is able to participate in N-cycling due to their atmospheric N-fixing capacity through interactions with Rhizobia bacteria^75^. Previously, it was observed that NAGase was essential for N mineralization. There may be a strong negative interaction because there are higher amounts of NH_4_^+^ and NO_3_^−^ and the mineral N stops the production of the NAG^67,76^. However, NAGase, Phase, and urease also showed a significant relationship with MBP. Enzymatic activities in the soil may act as an indicator for the presence of organic substances. Enzymatic processes that interact with the community of microbes have the potential to break down SOM and improve nutrient availability. BG enzyme activities have a significant and also show a significant negative relationship with TN. The correspondence with soil C was strongest for the N-acquire enzyme (NAG) and lower for C-acquire enzymes. Applying fertilizer can change the microbial population system by greatly increasing the number of absular mycorrhizal fungi. These fungi take carbon from the host plants and provide inorganic nutrients to the plants.

Table 9 demonstrates that the planting of chickpea cultivars did not significantly interact with Olsen P and Phase activity, but there was a relationship between soil P and MBP. A greater amount of soil MBP leads to an increase in the available P content of the soil; hence, soil microbes’ microorganisms efficiently uptake orthophosphate from the soil solution, and P can also be secreted from the pool of MBP due to microbial turnover^77^. According to^78^ both factors exhibited major negative interactions with each other in their studies where P fertilizers were applied and in naturally fertile soil. Positive associations between soil P and phase activities play a great role in P cycling; however, the relations among soil P and phase activities are more complex, which is not seen in the present. A massive group of soil microbes that can solubilize organic P and microbial biomass is a vital factor for measuring the activity of the enzyme in an agricultural environment, which is mainly related to increased metabolic activities^79^.

There was a strong relationship between phase enzyme and soil P and a significant negative relationship between MBP and soil K during pea plantations. Previous research supports the current study by showing that soil respiration developed after long-term P addition, suggesting that microbial activities are higher with the occurrence of P addition, which allows quick transformation of the soil available. It is observed that phosphate activities are essential for soil P metabolism, P legume species from root secretions, and plant P enhancement as well^80^. It is considered that P and K fertilizer amendments can alter the microbial community, and influences on the relative abundance of abuscular mycorrhizae fungi, which acquire C from their host plants, result in a negative relationship with soil K and MBP^81^. Soil microbial population, enzymatic activities, and MBP are essential factors in soil fertility status.

## Conclusion

The current study results indicated that P and K fertilization increased the nutrient accumulation capabilities of legumes. The microbial biomass phosphorus and soil potassium content increased in the T3 treatment (applied PK fertilizer together). While the K fertilization treatment is more effective for soil nutrient status and increases soil P availability. The application of fertilizer could influence soil properties and enzymatic activity. Current results suggest that P and K fertilization could be applied in different treatments, while combined PK fertilization can increase soil enzymatic activities and increase microorganisms' activities in soil, resulting in improvements to soil fertility and nutrient supply to the plant that could help to improve plant production. Future research needs to explain the role of different species of legumes in the structure of the community of soil microbes, directly measure the soil microorganism’s role which interacted with soil nutrient and the mechanisms underlying their correlations. Additionally, incorporating organic and inorganic fertilizers should be suggested as a superior nutrient management solution for intensive and sustainable green manure crop yields, which subsequently improves main crop yield. Furthermore, research needs to determine the MBP and microbe’s status in adding specific microorganisms (e.g., biological fertilizers).

## Data Availability

The datasets analyzed during this study are included in this manuscript.
